# Practical Eugenics

**Published:** 1913-07

**Authors:** 


### Practical Eugenics.

The women of Washington are organizing a society for the practical promotion of eugenics, a movement endorsed by Mrs. Woodrow Wilson, Mrs. William Jennings Bryan, Mrs. John Hays Hammond and other prominent women, all of whom will probably become members.

It has become the object of many society women there and their chief aim is to spread "educational eugenics" which will look to such subjects as:

Sex instruction for children.

The demand that marriageable men should have "not worldly capital only, but biological capital."

Bestowing on woman a voice in the selection of a mate.

Legislation against the marriage of persons physically unfit.

Segregation of the unfit.

A single standard of morals.

The homes of leading women have been thrown open for meetings relative to problems of eugenics.

The President's wife is known to have expressed herself as being strongly in favor of this phase of social reform.

Prominent in the organization will be Dr. Elnora Cuddeback Folkmar, who thus states the purposes of the proposed organization:

"It is time that the old puritanical ideas of modesty were abolished," she said. "It is time that boys and girls should be taken into our confidence with regard to those matters upon which their future happiness and welfare, as well as that of the race, depend.

"How much longer, I wonder, will we sit idly by and watch the propagation of blind, feeble-minded and epileptic children? How much longer will we permit men to wreck the lives of innocent and unsuspecting women?

"Believing that a thorough system of instruction and education in sex hygiene is the surest means of remedying present evils, the National Society for the the Promotion of Practical Eugenics will undertake an educational campaign by means of literature and illustrated lectures.

"It will strive to impress upon parents the necessity for plain speech and open dealing with their children. When our educational campaign is well under way in this city, the forming of eugenic societies in other cities will be the next logical step. Later we intend to get legislation which will prohibit the marrying of the unfit."

Supporting Dr. Folkmar in every move is Mrs. J. H. Hammond, one of the acknowledged leaders of Washington society. But Mrs. Hammond sounded a note of warning against too practical eugenics. Love always must play a part in perfect unions, she said.

"A mere physical union, made for the sake of producing perfect offspring, would not be a desirable ideal for individuals or nations," said said today. "The problems of eugenics will never be solved without that love between man and woman which is the cornerstone of the orthodox marriage and the orthodox home.

"But let us teach our boys and girls, our youths and maidens, the physical facts of life that make for better men, happier women and more perfect children."—*San Antonio Express.*

